# Biodegradable Zn-Cu-Fe Alloy as a Promising Material for Craniomaxillofacial Implants: An *in vitro* Investigation into Degradation Behavior, Cytotoxicity, and Hemocompatibility

**DOI:** 10.3389/fchem.2022.860040

**Published:** 2022-06-06

**Authors:** Yan Xu, Yichen Xu, Wentai Zhang, Ming Li, Hans-Peter Wendel, Jürgen Geis-Gerstorfer, Ping Li, Guojiang Wan, Shulan Xu, Tao Hu

**Affiliations:** ^1^ Center of Oral Implantology, Stomatological Hospital, Southern Medical University, Guangzhou, China; ^2^ State Key Laboratory of Oral Diseases and National Clinical Research Center for Oral Diseases, Department of Oral Prosthodontics, West China Hospital of Stomatology, Sichuan University, Chengdu, China; ^3^ Section Medical Materials Science and Technology, University Hospital Tübingen, Tübingen, Germany; ^4^ Key Laboratory of Advanced Technologies of Materials, Ministry of Education, School of Materials Science and Engineering, Southwest Jiaotong University, Chengdu, China; ^5^ Department of Materials Engineering, Sichuan Engineering Technical College, Deyang, China; ^6^ Department of Thoracic and Cardiovascular Surgery, Clinical Research Laboratory, University Hospital Tübingen, Tübingen, Germany; ^7^ State Key Laboratory of Oral Diseases and National Clinical Research Center for Oral Diseases, Department of Preventive Dentistry, West China Hospital of Stomatology, Sichuan University, Chengdu, China

**Keywords:** biodegradable metals, zinc, cytocompatibility, hemocompatibility, corrosion, implant, bone tissue engineering

## Abstract

Zinc-based nanoparticles, nanoscale metal frameworks and metals have been considered as biocompatible materials for bone tissue engineering. Among them, zinc-based metals are recognized as promising biodegradable materials thanks to their moderate degradation rate ranging between magnesium and iron. Nonetheless, materials’ biodegradability and the related biological response depend on the specific implant site. The present study evaluated the biodegradability, cytocompatibility, and hemocompatibility of a hot-extruded zinc-copper-iron (Zn-Cu-Fe) alloy as a potential biomaterial for craniomaxillofacial implants. Firstly, the effect of fetal bovine serum (FBS) on *in vitro* degradation behavior was evaluated. Furthermore, an extract test was used to evaluate the cytotoxicity of the alloy. Also, the hemocompatibility evaluation was carried out by a modified Chandler-Loop model. The results showed decreased degradation rates of the Zn-Cu-Fe alloy after incorporating FBS into the medium. Also, the alloy exhibited acceptable toxicity towards RAW264.7, HUVEC, and MC3T3-E1 cells. Regarding hemocompatibility, the alloy did not significantly alter erythrocyte, platelet, and leukocyte counts, while the coagulation and complement systems were activated. This study demonstrated the predictable *in vitro* degradation behavior, acceptable cytotoxicity, and appropriate hemocompatibility of Zn-Cu-Fe alloy; therefore, it might be a candidate biomaterial for craniomaxillofacial implants.

## Introduction

Craniomaxillofacial (CMF) areas present unique challenges to reconstructive surgery because of the complex bone morphology and stress distribution (Susarla et al., 2011). Metallic materials, in the form of mini-plates, mini-screws, and meshes, have been widely adopted for CMF implant applications due to the advantage of high mechanical strength (Schumann et al., 2013; Zwahlen, 2014). The fabrication and development of nanoscale metallic materials can improve their biological properties for bone tissue engineering (Zhang et al., 2020; Zhong et al., 2020; Fardjahromi et al., 2022). Conventionally, bioinert metallic materials, such as titanium-based alloy, cobalt-chromium-based alloy, and 316L stainless steel, have been used due to their excellent biocompatibility and high mechanical strength (Chen and Thouas, 2015). Nevertheless, bioinert metallic materials have several disadvantages. Regarding long-term clinical use, several adverse side effects have been documented, including infection, bone resorption caused by stress shielding, and even foreign-body host response (Alexander and Theodos, 1993; Hernandez Rosa et al., 2016). Furthermore, after tissue healing, an additional surgical procedure is required to remove these implants, probably leading to potential surgical risks (Busam et al., 2006; Schepers et al., 2011). In addition, most bioinert metallic materials lack appropriate biofunctionality (i.e., osteoinductivity and osteoimmunomodulation) to promote tissue regeneration and remodeling (Xie et al., 2020). Therefore, increasing research interests and clinical demands are shifting toward new metallic materials.

Biodegradable metals (BMs, also referred to as absorbable metals), i.e., magnesium (Mg), iron (Fe), zinc (Zn), and their alloys, refer to metals that degrade safely in a physiological environment and are considered promising materials as temporary medical implants (Zhao et al., 2017; Liu Y. et al., 2019; Han et al., 2019). Meanwhile, the applications of metal nanoparticles have recently been reported, as well as nanoscale metal-organic frameworks (Zhang et al., 2020). In the 19th century, Mg-based wires were first used as ligatures. Until now, Mg and Mg-based alloys have been widely investigated as biomedical implants due to their superior biocompatibility and appropriate mechanical properties (Witte, 2010; Rahman et al., 2020). Nonetheless, concerning Mg biodegradation, its rapid degradation rate with the massive accumulation of hydrogen around Mg-based implants could impede local tissue healing and reconstruction (Geis-Gerstorfer et al., 2011; Kraus et al., 2012). To overcome this drawback, Fe-based BMs have been proposed and investigated. Fe and Fe-based alloys have been considered absorbable biocompatible materials for osteosynthesis implants due to their high strength (Gorejová et al., 2019). Regarding biosafety of Fe-based materials, previous *in vivo* studies revealed no iron excess or organ damage caused by iron-based degradation products, indicating acceptable biocompatibility (Peuster et al., 2006; Waksman et al., 2008; Wu et al., 2013). According to ISO 10993-4, Fe-based materials exhibited excellent blood compatibility with a hemolysis rate below 5% as well as anti-platelet adhesion (Schinhammer et al., 2013; Verhaegen et al., 2019). However, some previous *in vivo* studies demonstrated that Fe-based implants within physiologic environments had a relatively slow degradation rate and formed insoluble degradation products, adversely affecting the local tissue remodeling (Pierson et al., 2012; Drynda et al., 2015).

Zinc plays an important role in physiological bone homeostasis and pathological bone turnover. Indeed, it has been found that zinc can stimulate osteoblast proliferation, differentiation, and mineralization. (O’Connor et al., 2020; Wang et al., 2021). Thus, Zn-based BMs have attracted ever-increasing attention for over one decade, mainly due to their superior biocompatibility and moderate degradation behavior (Katarivas et al., 2017; Zhang et al., 2021a; Li et al., 2021). Interestingly, Zinc-based nanoscale metal-organic frameworks have attracted a raising attention due to the toxicity of Zn^2+^ (Zhang et al., 2020). As the main degradation product of Zn-based metallic materials, the ionic Zn is regarded as an essential mineral of the human body, participating in multiple functional roles in metabolism and the immune system (O'Neill et al., 2018; Su et al., 2019) Based on standard corrosion potential, the potential of Zn (−0.77 V_SCE_) is between those of Mg (−2.37 V_SCE_) and Fe (−0.44 V_SCE_), indicating that the intrinsic biodegradability of Zn is between Mg and Fe (Zheng et al., 2017; Eliaz, 2019). Previous studies on the hemocompatibility of Zn and Zn-based alloys have demonstrated that they have acceptable hemolysis and platelet aggregation percentages (Kafri et al., 2018; Králová et al., 2021). Additionally, Zn-based alloys might be potential anticoagulation, due to their ability to slow prothrombin and partial thromboplastin times (Yin et al., 2019). In fact, previous *in vivo* investigations also demonstrated that Zn-based stent showed a steady degradation rate in the arterial environment, and systemic toxicity could be detected 1 year after implantation (Drelich et al., 2017; Yang et al., 2017).

Nonetheless, pure Zn as a CMF implant is not without its issues. The main shortcoming of pure Zn is its relatively poor mechanical strength and ductility, making it insufficient for CMF implant applications (Li et al., 2019b; Zhang et al., 2021a). Mechanical properties were improved by adding alloying elements such as Mg, Cu, Fe, Sr, etc., and/or thermomechanical treatment, including extrusion, rolling, forging, and annealing, to overcome this drawback (Li et al., 2018a; Sagasti et al., 2019; Capek et al., 2021). Furthermore, various novel alloy systems have been developed and investigated, including the Zn-Mg alloy, Zn-Ag alloy, and Zn-Cu alloy systems. Copper is a critical component and catalytic agent in many enzymes and proteins in the body, so it has various effects on human health. Furthermore, previous studies reported that the incorporation of Cu ions into biomaterial could positively impact healing mechanisms, particularly angiogenesis (Barralet et al., 2009; Kong et al., 2014). In addition, released Cu ion could promote the differentiation of mesenchymal stem cells and osteoblastic cells, which means that Cu-containing biomaterials have the potential to help accelerate bone regeneration (Rodríguez et al., 2002; Ewald et al., 2012). Due to copper’s biological activities and benefits, increasing research interests are shifting toward the development of new copper-containing biomaterials with improved cardioprotection and bone regeneration. Additionally, the Zn-Cu-Fe alloys with superior mechanical properties have been fabricated and investigated for potential biomedical implants (Yue et al., 2019; Yue et al., 2020; Zhang et al., 2021b). Nonetheless, the *in vitro* degradation behavior, cytocompatibility, and homonormativity should be further evaluated concerning the craniomaxillofacial area.

Compared with most-reported biodegradable Zn-based alloys, a hot-extruded Zn-Cu-Fe alloy with excellent mechanical properties has been developed (Zhang et al., 2021b). In this study, we first aimed to investigate the effect of serum on the *in vitro* degradation behavior of the Zn-Cu-Fe alloy using an immersion test under cell culture conditions. Furthermore, the cytotoxicity of the Zn-Cu-Fe alloy was evaluated by an extract test. Different bone-related cell lines, i.e., macrophages, venous endothelial cells, and osteoprogenitor cells, were used. In addition, the dynamic hemocompatibility of the Zn-Cu-Fe alloy was tested through a modified Chandler-Loop model. Finally, blood parameters of clinical relevance were analyzed, such as hematological parameters (erythrocytes, leukocytes, and platelets) and the related plasma markers.

## Materials and Methods

### Specimen Preparation

A Zn-0.5Cu-0.2Fe (wt%) alloy (denoted as Zn-Cu-Fe) was fabricated and cast from pure Zn ingot (99.99 wt%), Cu wire (99.9 wt%), and Fe wire (99.9 wt%) followed by a series of thermochemical treatments, as previously reported (Zhang et al., 2021b). In short, the elements were melted under 1-bar argon in a graphite crucible and then cast in a rectangular graphite mold. Next, homogenization was carried out in a furnace at 300°C by hot rolling at 200°C and annealing at 390°C for 15 min. After that, the as-rolled pure Zn and Zn-Cu-Fe sheets were cut into 23 × 7 × 1.5 mm size and reduced to 1.5 mm thickness for further immersion tests and cytotoxicity evaluation. Also, the specimens were cut into strips measuring 50 × 7 × 1.5 mm for hemocompatibility tests. Before all the tests, the specimens were mechanically ground with silicon carbide abrasive paper up to grit 600. Next, the samples were ultrasonically cleaned in absolute ethanol for 10 min and immediately disinfected under ultraviolet radiation for at least 1 h in a workbench.

### Immersion Test

A semi-static immersion test was performed for pure Zn and Zn-Cu-Fe alloy. Hank’s balanced salt solution (HBSS) was used in the presence or absence of fetal bovine serum (FBS, ExCell Bio, Shanghai, China) as two different immersion media to evaluate the influence of serum on long-term degradation. Table 1 lists the compositions of the immersion media compared to the human extracellular fluid (Schille et al., 2011; Jung et al., 2015; Li et al., 2019a). Each specimen was individually weighed with a sensitivity of 0.1 mg using a digital steelyard (Q125, Sartorius AG, Germany). Next, five specimens per group were immersed in the HBSS with or without FBS, respectively. The surface area-to-solution volume ratio was set to 1.0 cm^2^/ml according to ISO 10271: 2011 (International Organization for Standardization (ISO), 2011). Furthermore, the samples were incubated under cell culture conditions (37°C, 5% CO_2_, 95% humidity) for 21 days to simulate physiologic conditions. The immersion media were refreshed every 48 h to simulate a semi-static immersion test. At the end of the immersion period, one sample per group was selected to analyze the visible insoluble products on surfaces under a scanning electron microscope (SEM) equipped with an energy dispersive x-ray (EDS) spectrometer at 10 kV (Gemini 300, Zeiss, Germany). In addition, degradation products on the material surfaces were removed by glycine (NH_2_CH_2_COOH) (250 g/L) for 10 min, according to ISO 8407: 2009 to determine degradation rates and modes (International Organization for Standardization(ISO), 2009b). Afterward, the degradation rate (DR) was measured by weight loss using the following equation: DR = ΔW/(D × T), where W is the weight loss before immersion and after removing degradation tests (μg) A is the surface area (cm^2^), and T is the immersion time (day). In addition, specimen surfaces were further analyzed by SEM-EDS after removing the degradation products.

**TABLE 1:** The main compositions of DMEM, McCoy’s 5A, and FBS, compared to the human extracellular fluid.

Composition	Human Extracellular Fluid		HBSS	FBS	DMEM
	Blood Plasma	Interstitial Fluid			
Inorganic ions (mM)					
Na^+^	142.0	139.0	141.2	137.0	127.3
K^+^	4.2	4.0	5.8	11.2	5.3
Mg^2+^	0.8	0.7	0.7	n.m	0.8
Ca^2+^	1.3	1.2	1.3	3.4	1.8
Cl^−^	106.0	108.0	144.8	103.0	90.8
SO_4_^2-^	0.5	0.5	0.2	n.m	0.8
HPO_4_^2-^	2.0	2.0	0.6	n.m	0.9
HCO_3_^−^	24.0	28.3	4.2	n.m	44.1
Organic components					
Protein	1.2 (mM)	0.2 (mM)	—	38.0 (g/L)	—
Glucose (mM)	5.6	5.6	5.6	6.9	4.5
Amino acids	2.0 (mM)	2.0 (mM)	—	n.m	1.6 (g/L)
Concentrations of buffering agents (mM)					
HCO_3_^−^	24.0	28.3	4.2	n.m	44.1
HPO_4_^2-^	2.0	2.0	0.6	n.m	0.9
HPr	16.0–18.0	—	—	n.m	—
Tris-HCl	—	—	—	n.m	25.0
Total	42.0–44.0	30.3	4.8	n.m	70.0
References	Li et al. (2019a)	Li et al. (2019a)	Li et al. (2019b)	Li et al. (2019a)	Li et al. (2019b)

### Cytotoxicity Test

An extract test was used for the cytotoxicity evaluation of the Zn-Cu-Fe alloy based on ISO 10993-5: 2009 (International Organization for Standardization(ISO), 2009a) and ISO 10993-12: 2012 (International Organization for Standardization (ISO) (2011)). Also, a titanium-based alloy (Ti-6Al-4V) was used as a negative control, and pure copper (Cu) was used as a positive control. Three different types of bone-related cell lines, i.e., murine macrophages (RAW264.7), human umbilical vein endothelial cells (HUVEC), and mouse preosteoblast cell line (MC3T3-E1), were used. RAW264.7 and HUVEC cells were cultured in Dulbecco’s Modified Eagle’s Medium (DMEM, Thermo Fisher Scientific, United States) with 10% FBS, 1% penicillin/streptomycin, and 1% GlutaMAX (Life Technologies, United Kingdom). MC3T3-E1 cell was cultured in Alpha Minimum Essential Medium (α-MEM, Thermo Fisher Scientific, United States) with 10% FBS, 1% penicillin/streptomycin. The specimens were immersed in a cell culture medium with a surface area-to-extraction medium ratio of 1.25 cm^2^/ml for 72 h under standard cell culture conditions. Afterward, the extracts were gradient-diluted to 50, 25, and 12.5% extracts, respectively.

Cytocompatibility was qualitatively evaluated by the cell membrane integrity and quantitatively analyzed by determining metabolic activity and detecting lactate dehydrogenase (LDH) release. The cells were seeded at a density of 3 × 10^4^ cells/cm^2^ and pre-cultivated overnight. Cell culture media were replaced by sample extracts for 24 h. Subsequently, the cells were stained by live/dead staining containing a calcein acetoxymethyl (Calcein-AM) reagent and propidium iodide (PI), following the manufacturer’s instructions. Cell morphology and viability were observed under an inverted fluorescence microscope (DMi8, Leica Microsystems GmbH, Germany). A tetrazolium-based assay (CCK-8, Dojindo, Japan) and an LDH assay (Beyotime Biotechnology, China) were used to determine the results quantitatively. Briefly, after incubation with the extracts, the cell supernatants were collected and incubated with LDH reaction reagent as per the manufacturer’s instructions. The absorbance data were collected using a microplate reader (SpectraMax plus384, Molecular Devices, China) at a wavelength of 490/600 nm. Meanwhile, the CCK-8 reagent was added to each well for 2 h. Next, the absorbance data were detected at 450 nm. Finally, the relative metabolic activity and LDH release were calculated, as reported before (Jung et al., 2015; Li et al., 2019a).

### Chandler-Loop Experiments

**FIGURE 1:**
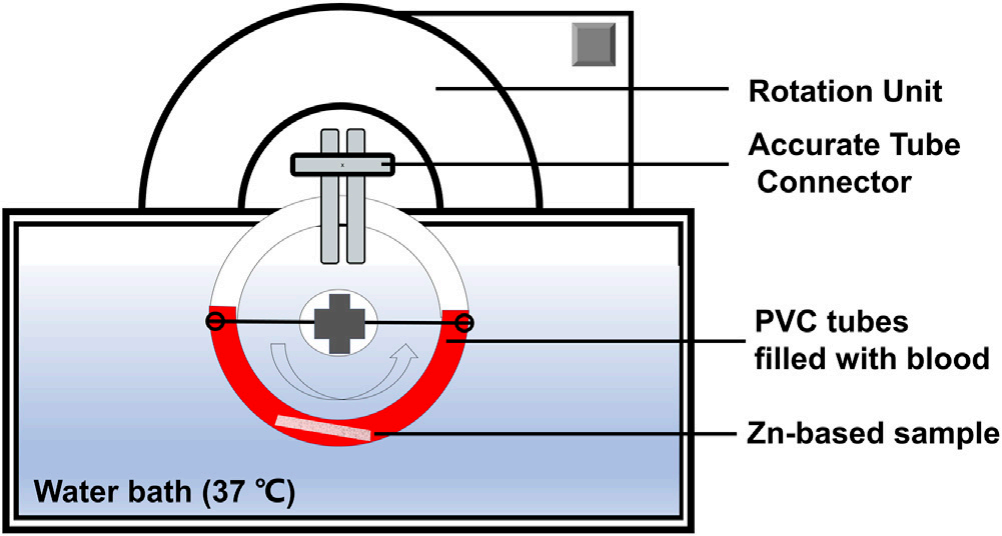
Schematic representation showing the modified Chandler-Loop model.

Hemocompatibility was evaluated using a modified Chandler-Loop model for 1 h, according to ISO 10993-4: 2017 (International Organization for Standardization(ISO), 2017). As shown in Figure 1, a closed-loop circulation model consists of a controlled rotation unit with polyvinyl chloride (PVC) loops in a thermostated water bath (37°C) to mimic blood physiologic circulation. Four specimens per group were individually placed into the PVC loop (ECC-noDOP 3/8”, Raumedic AG, Rehau, Germany). Before the tests, human peripheral blood samples were collected, as we previously reported (Zhang et al., 2021a). Subsequently, each PVC tube was filled with 30 ml of fresh human blood. The rotation rate was set vertically at 30 rpm in the water bath at 37°C. After 60 min of circulation, the blood was collected for hemocompatibility evaluation in each. In addition, the blood sample without circulation was set as “baseline”, and the blood without specimen after 1 h of circulation was used as a “control”.

After circulation, clinical blood parameters were analyzed from three aspects, namely erythrocytes, leukocytes, and platelets. Erythrocytes were counted using an automated hematology analyzer (ABX Micros 60, Montpellier, France). Also, hemolysis was measured by a colorimetric assay for free plasma hemoglobin. Hematocrit and hemoglobin values were determined by a photometric test. For the platelets and induced coagulation, the platelet counts were determined. Beta-thromboglobulin (β-TG) levels were measured to determine platelet activation following the manufacturer’s instructions (Diagnostica Stago/Roche, Mannheim, Germany). Also, thrombin-antithrombin complex-III (TAT) was detected for coagulation (Siemens Healthcare, Marburg, Germany). Concerning the leukocytes and the related inflammation, the cell counts were determined. Polymorphonuclear elastase (PMN-elastase) secretion was assessed by an enzyme-linked immunosorbent assay (Demeditec Diagnostics GmbH, Kiel, Germany) according to the manufacturer’s instructions. In addition, the product of terminal pathway complement activation (SC5b-9) was measured (Osteomedical GmbH, Bünde, Germany) to evaluate complement activation.

### Statistical Analysis

The degradation rate data were tested by a two-way analysis of variance (ANOVA) with serum and alloying as independent factors, followed by Tukey’s multiple comparisons. Regarding the cytotoxicity results, an unpaired Student’s t-test was used to evaluate significant differences between Zn and Zn-Cu-Fe. Furthermore, the results of hemocompatibility analysis were measured by one-way ANOVA between different groups, followed by Tukey’s test for the subsequent multiple comparisons. The GraphPad Prism software (V6.01, GraphPad Software, San Diego, CA, United States) was used for all statistical analyses at a significance level of α = 0.05.

## Results

### *In vitro* Degradation Behavior

**FIGURE 2:**
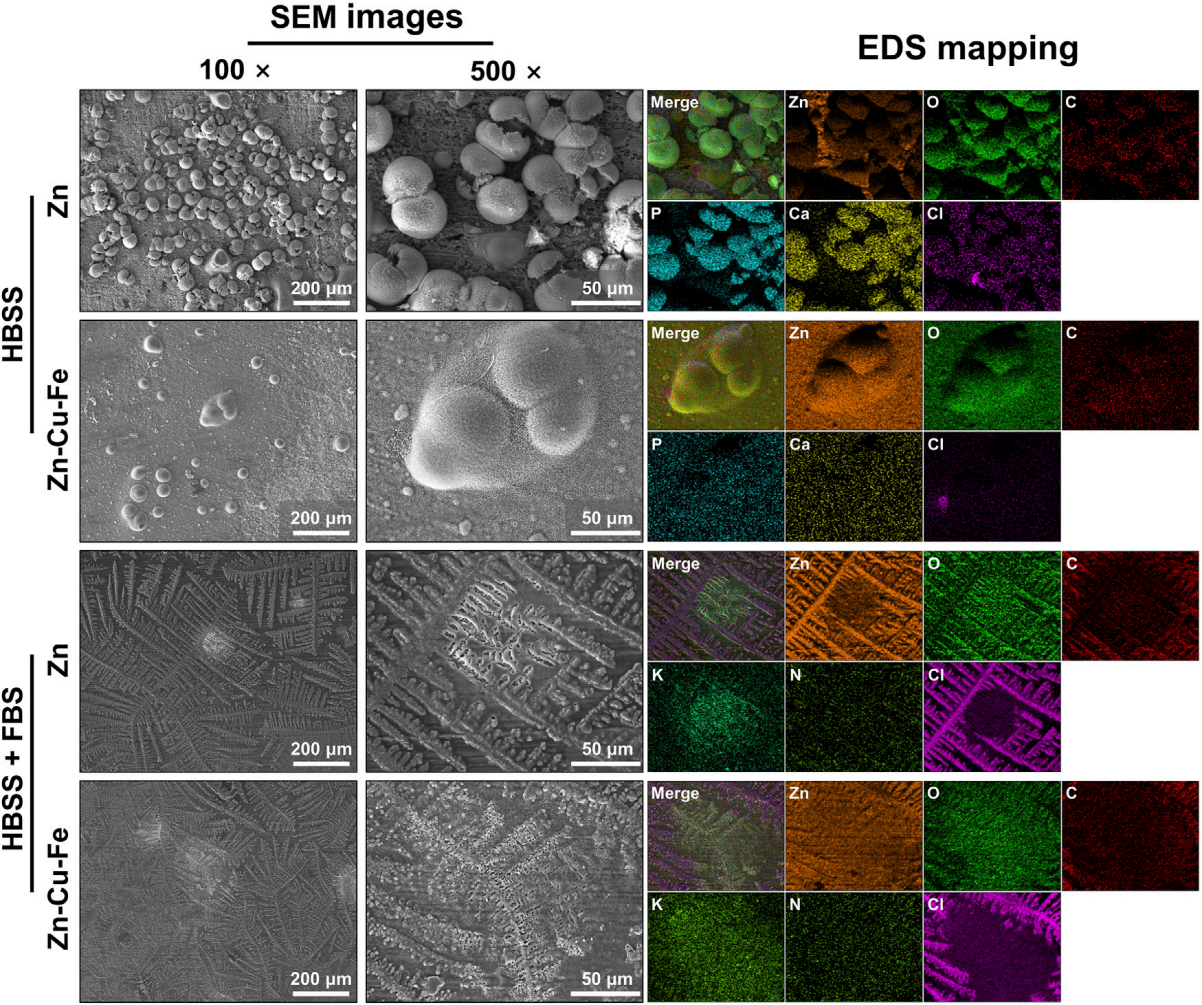
Surface characterization of pure Zn and Zn-Cu-Fe alloy after immersion in HBSS with or without FBS under cell culture conditions for 21 days. Representative SEM images show degradation products on the surfaces after the immersion test (magnification: 100 × and 500 ×) at 15 kV. The results of EDS mapping indicate the elemental composition of degradation products.

Figure 2 presents the degradation products and their elemental composition on specimen surfaces after 21 days of immersion in the HBSS with or without FBS. The degradation precipitates dispersed on sample surfaces showed obviously different degradation morphologies in the HBSS in the absence or presence of FBS. Regarding samples in HBSS without FBS, SEM images at low magnification revealed the distribution of some spherical-like degradation particles and thick degradation layers covering the whole surfaces. Samples in HBSS with FBS showed tiny conifer-like degradation precipitates spreading over the whole surfaces, but the grinding scratches could still be observed at high magnification (500 ×). No apparent differences in surface morphologies were observed between Zn and Zn-Cu-Fe in the same simulate body fluid. However, the concentration of degradation products on the Zn surface in the HBSS was obviously higher than the counterpart Zn-Cu-Fe alloy. Additionally, EDS analysis showed that the main elemental components of the degradation products on sample surfaces in HBSS without FBS were Zn, O, C, P, Ca, and Cl, indicating the formation of phosphate and carbonate corrosion products. Interestingly, degradation products on sample surfaces in HBSS with FBS had a similar elemental composition (Zn, O, C, and Cl) along with N and K elements, indicating that the organic product layers from the serum were formed on the surfaces.

**FIGURE 3:**
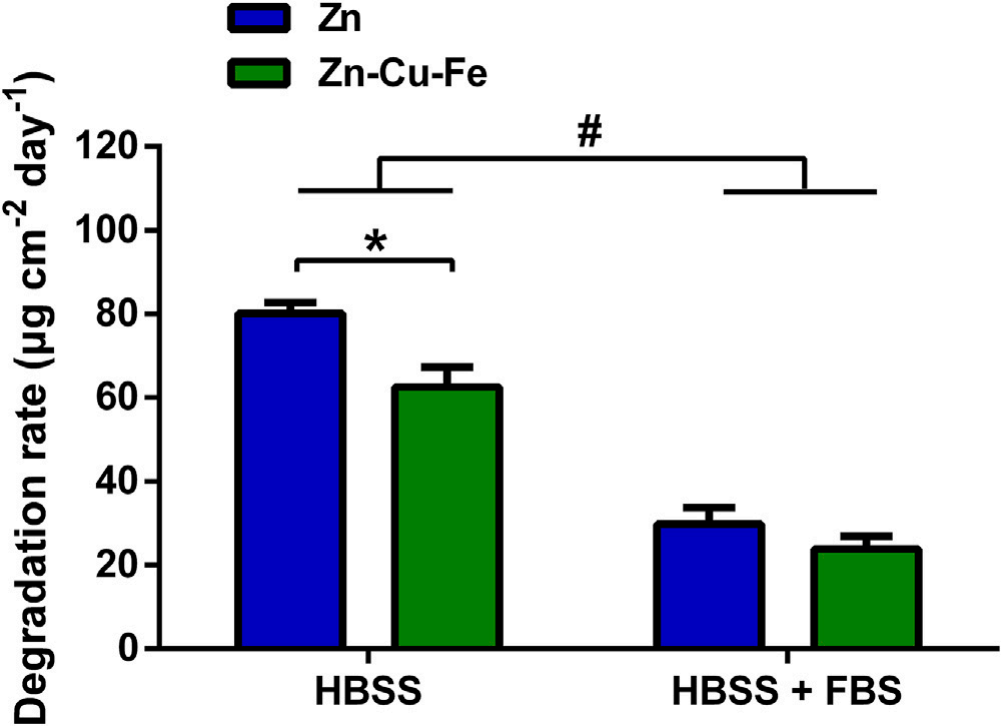
The *in vitro* degradation rates of Zn and Zn-Cu-Fe were calculated by weight loss (n = 4). The hashtag indicates a significant difference (*p* < 0.05) between HBSS and HBSS with FBS, determined by two-way ANOVA. The asterisk represents a significant difference in Zn and Zn-Cu-Fe in the HBSS (*p* < 0.05) by two-way ANOVA followed by Tukey’s multiple comparisons.

As shown in Figure 3, the degradation rates of pure Zn and the Zn-Cu-Fe alloy were calculated in different solutions. A two-way ANOVA was conducted to examine the effects of serum and alloying on the degradation rates. The serum had statistically significant interactions with different materials concerning its effect on degradation rate (F_(1, 12)_ = 9.934, *p* = 0.008), confirmed by two-way ANOVA. Each main effect also exhibited significant impact: serum (F_(1, 12)_ = 582.1, *p* < 0.0001) and alloying (F_(1, 12)_ = 40.640, *p* < 0.0001). Notably, regarding the HBSS groups, the degradation rate of the pure Zn (80.10 ± 2.59 μm cm^−2^ day^−1^) was significantly higher than that of the Zn-Cu-Fe alloy (62.54 ± 4.84 μm cm^−2^ day^−1^, *p* = 0.001). However, for the groups of HBSS with FBS, Tukey’s multiple comparison tests revealed no significant differences between pure Zn and (29.83 ± 3.91 μm cm^−2^ day^−1^) Zn-Cu-Fe alloy (23.89 ± 2.98 μm cm^−2^ day^−1^, *p* = 0.158). Taken together, higher degradation rates of specimens were observed in HBSS. Also, there were no obvious differences in degradation rates in HBSS with serum between Zn and Zn-Cu-Fe.

**FIGURE 4:**
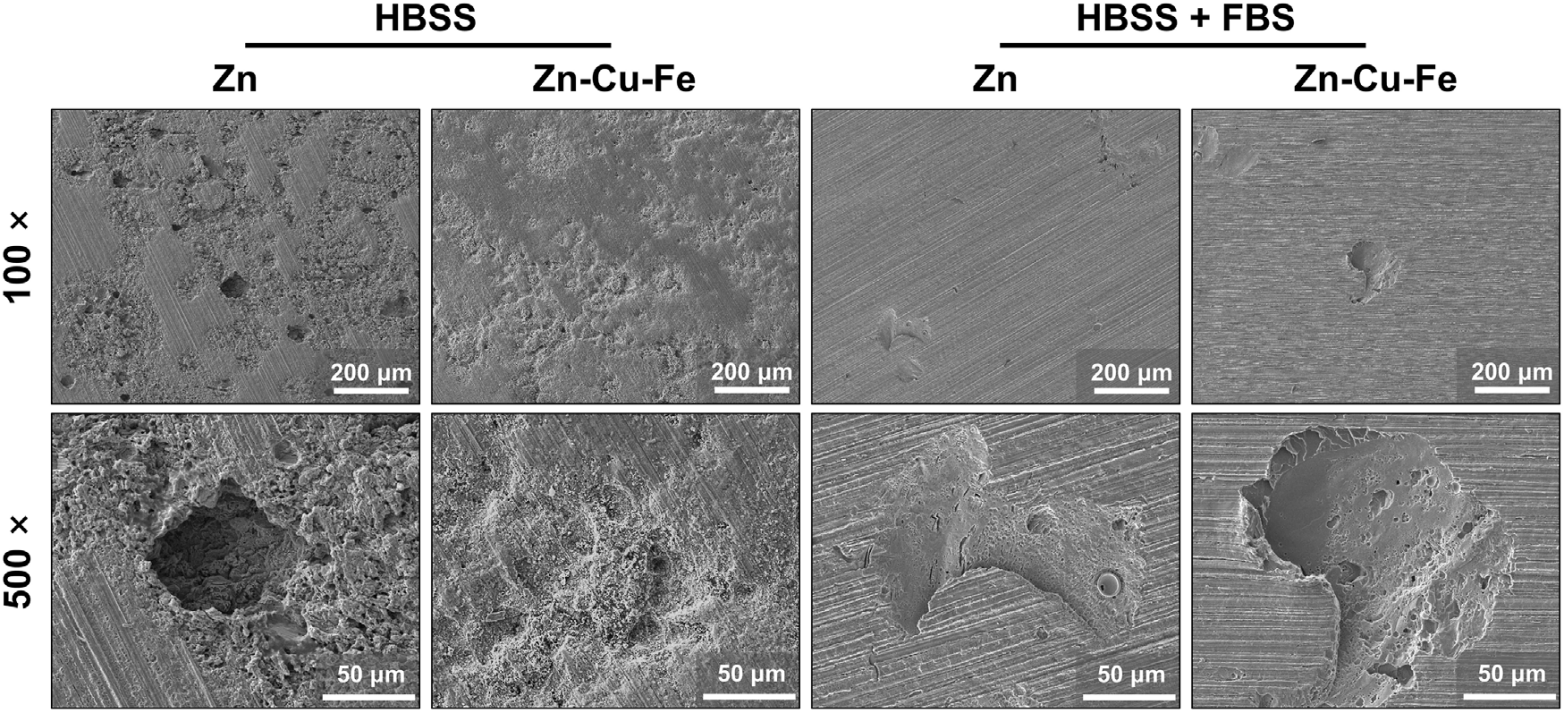
Representative SEM images of pure Zn and Zn-Cu-Fe alloy after removing degradation products (low magnification: 100 × and high magnification: 500 ×).

Figure 4 illustrates the corroded morphologies of Zn and Zn-Cu-Fe after removing the degradation products from the surfaces. Pure Zn exhibited corrosion morphology with large pits and extensive localized corrosion after immersion with HBSS. Additionally, the Zn-Cu-Fe showed a relatively uniform degradation morphology in HBSS, with fewer pits. In contrast, Zn and Zn-Cu-Fe immersed in HBSS with FBS exhibited milder and more uniform degradation, and grinding scratches remained on the surfaces. Only a few localized corrosion spots could be found on both surfaces.

### Cytocompatibility Evaluation

**FIGURE 5:**
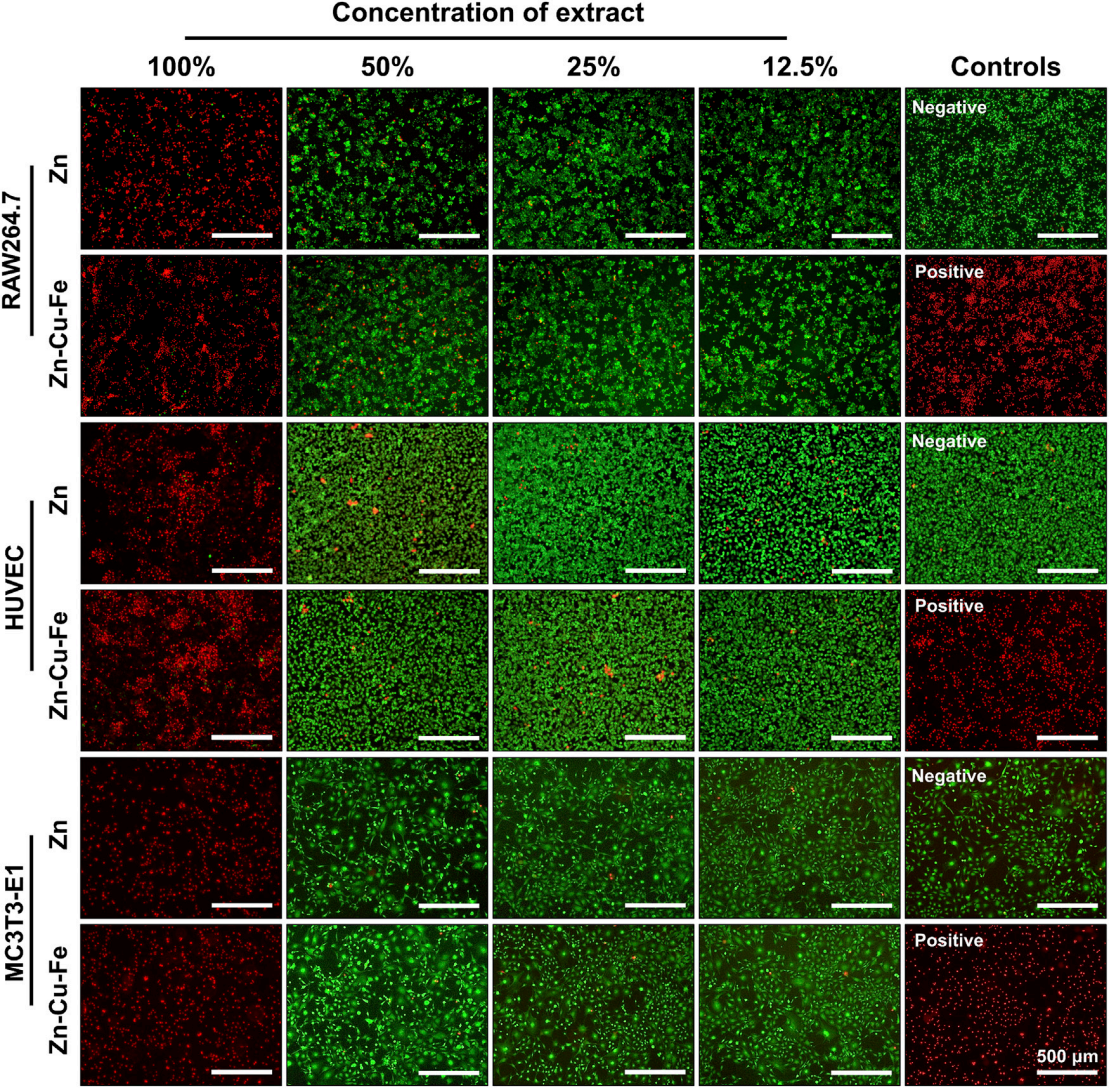
Representative fluorescence images of RAW264.7 cells, HUVEC cells, and MC3T3-E1 cells exposed to sample extracts for 24 h. Ti-based alloy extract was used as a negative control, with Cu extracts as a positive control. Green fluorescence represents viable cells with membrane integrity stained by calcein AM, and red fluorescence indicates apoptotic cells stained by EB.

The effects of sample extracts on cell membrane integrity were qualitatively determined by live/dead fluorescence staining, as illustrated in Figure 5. Three different cells exposed to sample extracts had consistent cellular responses. In the original undiluted extracts, almost all the cells were round-shaped and fluorescent-stained, consistent with the positive control, indicating that the apoptotic cells occurred in 100% of sample extracts. In contrast, cells exposed to the diluted sample extracts exhibited predominantly spindle-shaped cell morphology with green fluorescence, with only limited red fluorescent staining, consistent with the negative controls. This finding indicates that the cytotoxic effects decreased after diluting the sample extracts.

**FIGURE 6:**
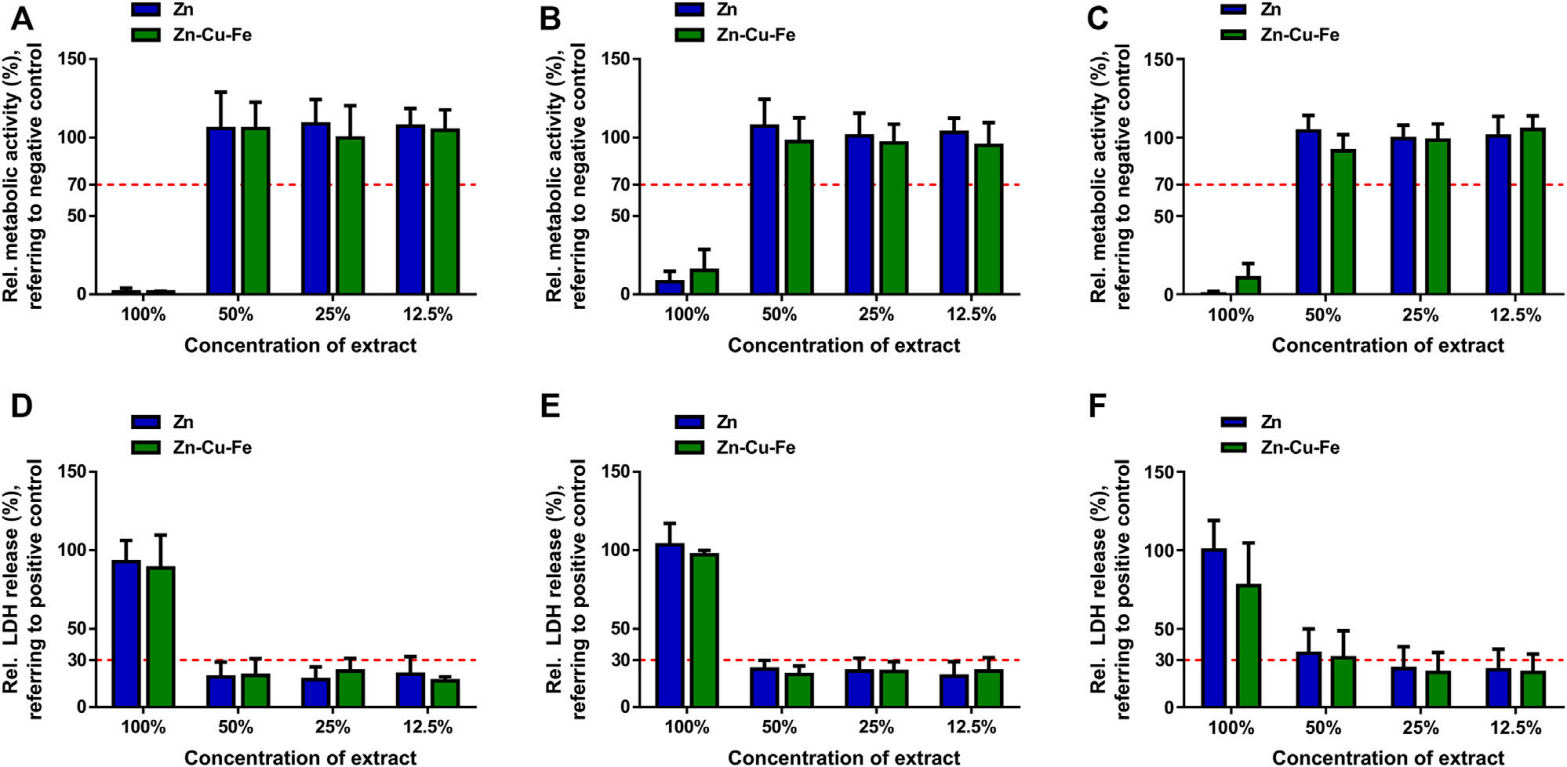
Quantitative results of cytotoxicity evaluation for pure Zn and Zn-Cu-Fe alloy. Relative metabolic activities of RAW264.7 cells **(A)**, HUVEC cells **(B)**, and MC3T3-E1 cells **(C)** exposed to sample extracts for 24 h. The red dashed line (70% of the negative control) stands for the cut-off level between non-toxic and toxic effects. Relative LDH release of RAW264.7 cells **(D)**, HUVEC cells **(E)**, and MC3T3-E1 cells **(F)** cultured in sample extracts for 24 h. The red dashed line (30% of the positive control) represents the cut-off between toxic and non-toxic results.

The relative metabolic activity and LDH release were further measured to determine cytocompatibility quantitatively. Figures 6A–C depict the relative metabolic activities of RAW264.7 cells, HUVEC cells, and MC3T3-E1 cells cultured in the Zn and Zn-Cu-Fe extracts. In undiluted extracts, lower metabolic activities (<20%) could be observed. However, the diluted extracts showed that the relative metabolic activities significantly increased to >70% of the negative control, indicating non-cytotoxic effects. Furthermore, relative LDH releases exposed to sample extracts were determined, as illustrated in Figures 6D–F. Three different cells cultured in 100% sample extracts showed much higher LDH release, corresponding to the positive control. In contrast, cells exposed to the diluted extracts showed a much lower LDH release, <30% of the positive control, considered non-toxic effects. Notably, regarding the respective extract, there were no statistically significant differences in cell metabolic activity and LDH release between Zn and Zn-Cu-Fe groups (*p* > 0.05), based on an unpaired Student’s t-test. Therefore, the overall tendency demonstrated that apparent cytotoxic effects were observed in undiluted sample extracts while the toxic effect decreased significantly after the sample extracts were diluted.

### Hemocompatibility Evaluation

**FIGURE 7:**
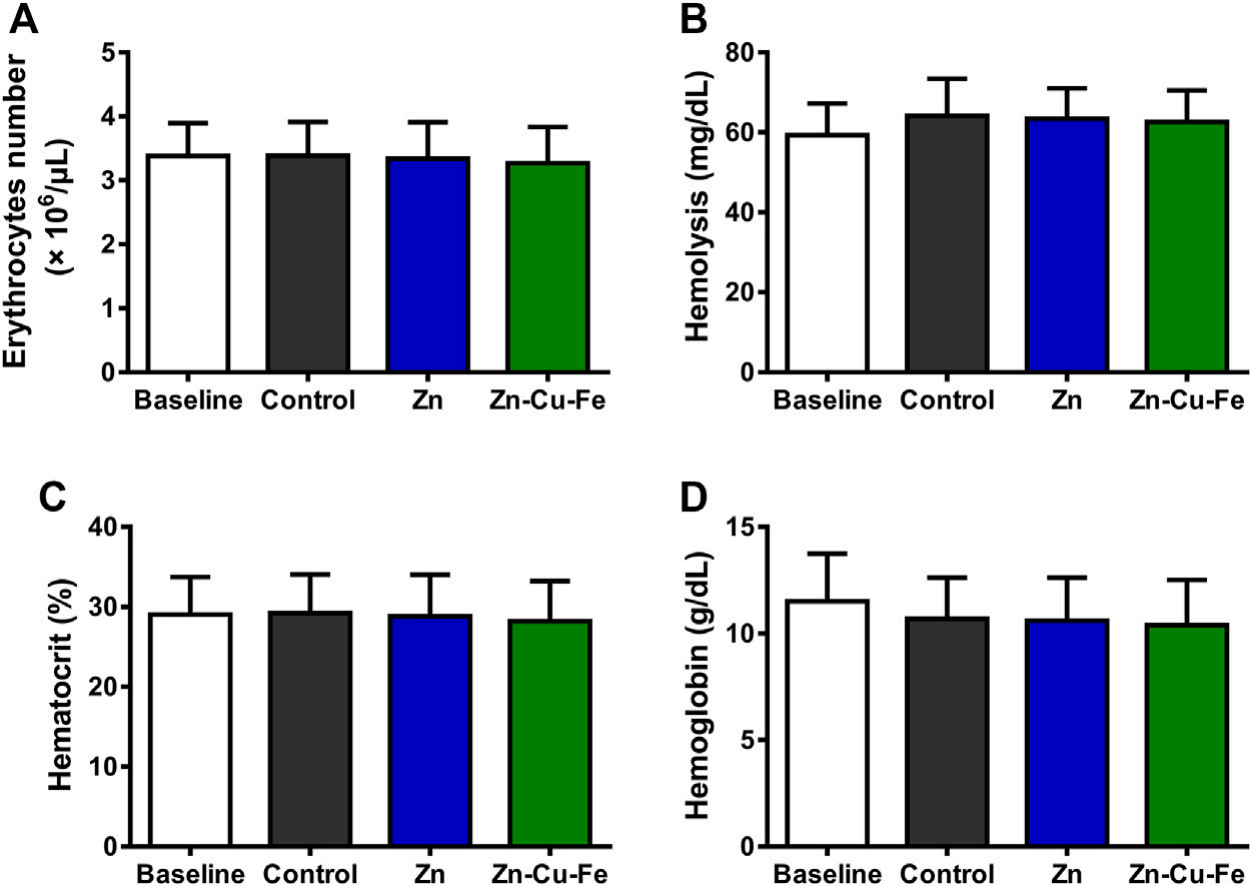
Hematological parameters were analyzed before (baseline) and after 60 min of circulation using the Chandler-Loop model: **(A)** the number of erythrocytes (10^6^/μL), **(B)** hemolysis calculated by the amount of cyan hemoglobin (mg/dl), **(C)** hematocrit values (%), and **(D)** hemoglobin concentrations (g/dl). Blood in the empty tubing after circulation was considered a control.

Human blood contact with Zn and Zn-Cu-Fe was evaluated before and after circulation in the Chandler-Loop model. As shown in Figure 7A, the applied statistics indicated no significant differences in erythrocyte counts between the different groups (F_(3, 12)_ = 0.039, *p* = 0.988). Meanwhile, hemolysis values by the amount of cyanhemoglobin showed no significant differences (F_(3, 12)_ = 0.273, *p* = 0.843) between the groups, determined by the ANOVA (Figure 7B). As illustrated in Figures 7C,D, there were no significant differences in hematocrit values (F_(3, 12)_ = 0.031, *p* = 0.992) and hemoglobin concentrations (F_(3, 12)_ = 0.216, *p* = 0.883) between the different groups. Therefore, these findings indicated that Zn and Zn-Cu-Fe did not adversely affect the erythrocyte function.

**FIGURE 8:**
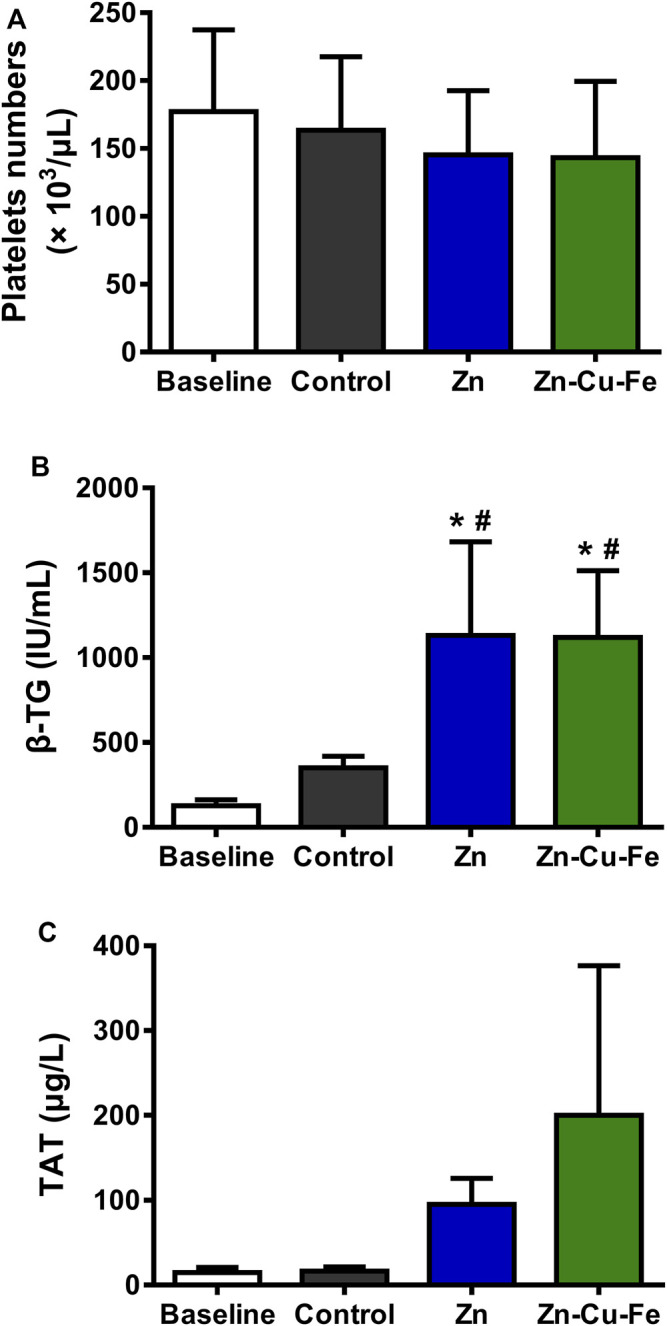
The number of platelets and plasma markers after 60 min of exposure to blood using the Chandler-Loop model: **(A)** the number of platelets (10^3^/μL), **(B)** β-thromboglobulin release (IU/ml), and **(C)** thrombin-antithrombin III level (ug/L). * and # indicate *p* < 0.05 based on one-way ANOVA followed by post hoc Tukey’s test, compared to the baseline and controls, respectively.

After 60 min of circulation, platelets and the related plasma markers were analyzed. Although the number of platelets exposed to Zn and Zn-Cu-Fe had slightly decreased, no significant differences could be observed between different groups (F_(3, 12)_ = 0.347, *p* = 0.791), as shown in Figure 8A. However, the concentration of β-TG was regarded as an efficient marker of platelet activation (Figure 8B). ANOVA was used to confirm statistically significant differences in β-TG concentration (F_(3, 12)_ = 9.288, *p* = 0.002). Post hoc pairwise comparisons indicated that the β-TG value of the control (350.9 ± 67.87 IU/ml) was significantly lower than those of Zn (1,131 ± 552.0 IU/ml, *p* = 0.032) and Zn-Cu-Fe (1,120 ± 393.6 IU/ml, *p* = 0.034), respectively. Furthermore, the TAT complex as a plasma marker was used to detect coagulation activation (Figure 8C). ANOVA confirmed statistically significant differences in TAT levels between different groups (F_(3, 12)_ = 3.811, *p* = 0.039). However, post hoc pairwise comparisons showed no significant differences between different groups (*p* > 0.05), probably due to the limited sample size and high deviations.

**FIGURE 9:**
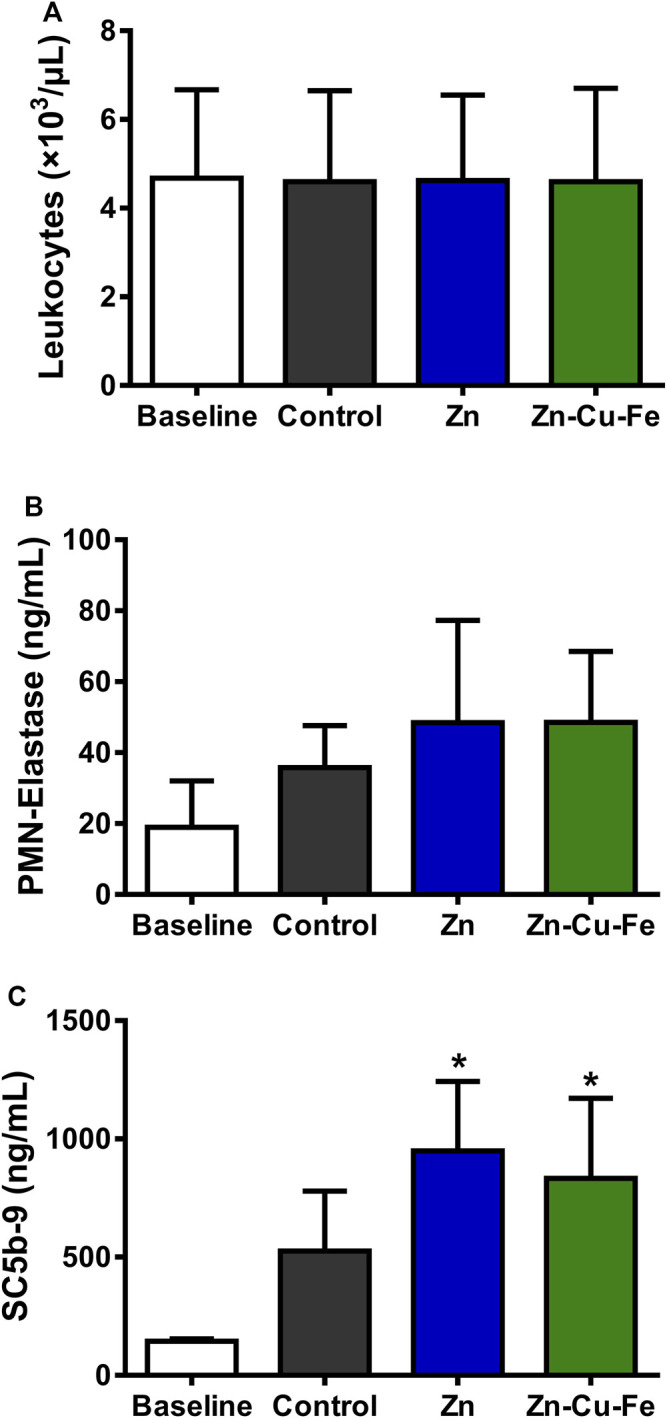
The number of leukocytes and plasma markers after 60 min of exposure to blood using the Chandler-Loop model: **(A)** the number of leukocytes (10^3^/μL), **(B)** the amount of PMN-elastase (ng/ml), and **(C)** the complement factor SC5b-9 (ng/ml). * indicates *p* < 0.05 according to one-way ANOVA followed by post hoc Tukey’s test, compared to the baseline.

As illustrated in Figure 9A, the number of leukocytes was not significantly different between different groups after 60 min (F_(3, 12)_ = 0.001, *p* > 0.999). Moreover, the PMN-elastase level indicated the amount of the unbound elastase complexed with the 1-proteinase inhibitor released after leukocyte activation. Figure 9B shows no significant differences in PMN-elastase levels between the different groups (F_(3, 12)_ = 2.041, *p* = 0.162). Moreover, plasma protein SC5b-9 was a marker of the inflammatory reaction following complement activation by biomaterials. As illustrated in Figure 9C, significant differences were detected in SC5b-9 values between different groups (F_(3, 12)_ = 7.826, *p* = 0.004). Specifically, Tukey’s multiple comparisons showed significantly higher values in the Zn (949.4 ± 293.2 ng/ml, *p* = 0.004) and Zn-Cu-Fe (832.7 ± 339.2, *p* = 0.012) groups than the baseline (144.2 ± 10.11 ng/ml).

## Discussion

An ideal degradable biomaterial for craniomaxillofacial implants should have the following properties: 1) satisfactory biological safety, with no carcinogenicity, teratogenicity, and adverse side effects on the tissue; 2) sufficient mechanical properties for load-bearing; 3) gradual degradation rates, with the body’s ability to metabolize the degradation products safely; 4) appropriate bioactive properties to promote tissue regeneration and reduce inflammation; and 5) easy processing and sterilization. Therefore, a hot-extruded Zn-Cu-Fe alloy was used in this study to evaluate its *in vitro* degradation behavior, cytotoxicity, and dynamic hemocompatibility.

### *In vitro* Degradation Behavior of Zn-Cu-Fe

Predicting *in vivo* degradation behavior is of considerable significance because biodegradability can determine the mechanical integrity and related biocompatibility. Nonetheless, a standardized degradation evaluation for biodegradable Zn-based metals is still lacking. Therefore, considering the potential clinical applications, the Zn-based implants are placed in a blood-filled implant site, which directly affects the corrosion behavior. In this study, to mimic the bone implantation environment, the fetal bovine serum was used as an *in vitro* model of blood. Additionally, the immersion test was performed under standard cell culture conditions to verify that the experimental set-up is similar to the physiological environment (Johnston et al., 2017; Gonzalez et al., 2018).

**FIGURE 10:**
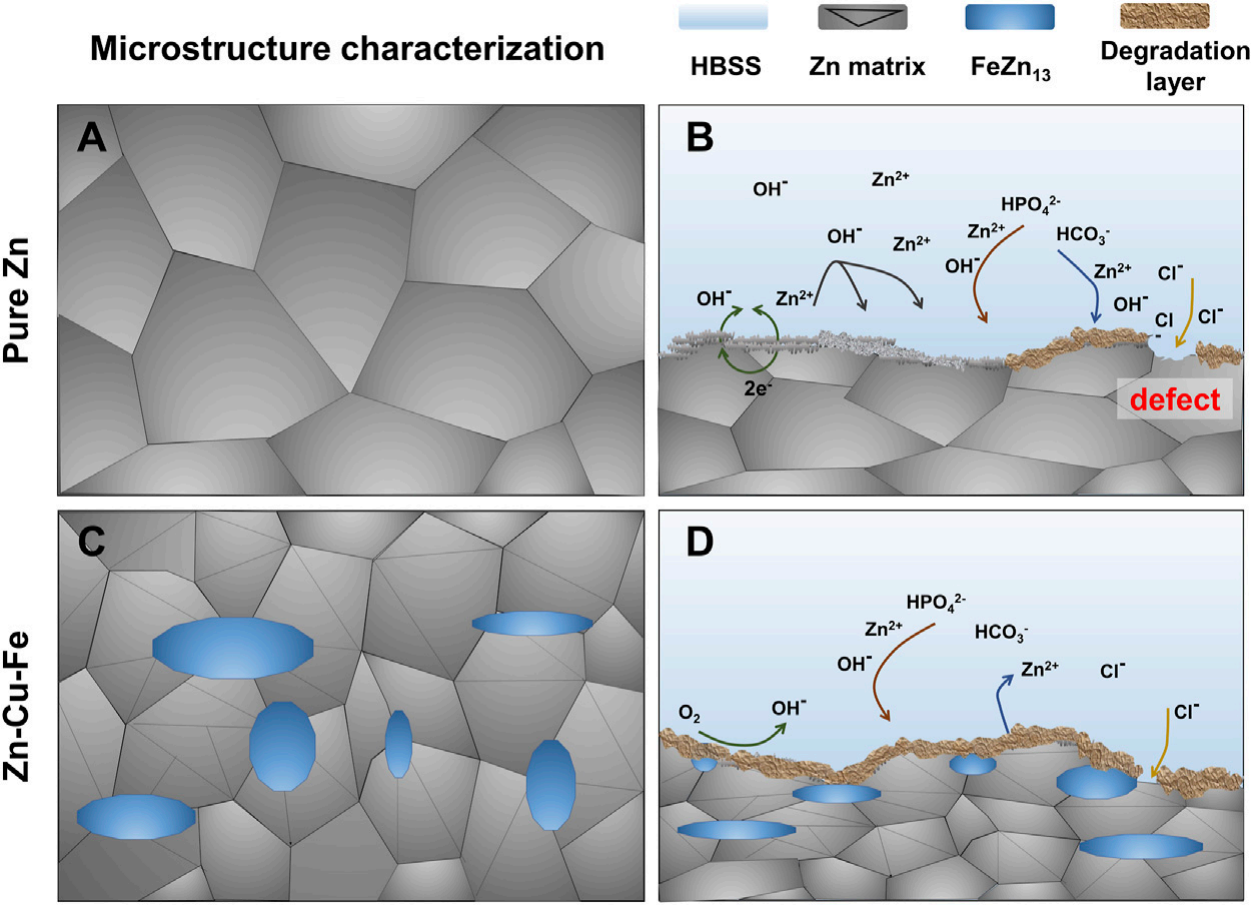
Schematic illustration showing the degradation behavior of Zn and Zn-Cu-Fe alloy. Microstructure characterization of the pure Zn **(A)** and Zn-Cu-Fe alloy **(B)**; the potential degradation mechanism of the pure Zn **(C)** and Zn-Cu-Fe alloy **(D)**.

In this study, the *in vitro* degradation behavior of Zn-Cu-Fe alloy in the HBSS with and without FBS was investigated compared to the Zn degradation behavior. According to the results, the following Zn degradation mechanism of the alloy in HBSS with and without FBS could be deduced, as illustrated in Figure 10. When Zn and Zn-Cu-Fe are immersed in HBSS (slightly alkaline solution, Table 1), anodic dissolution of the metal and the cathodic reduction of oxygen can occur, referring to Eq. 1 and Eq. 2 (Bowen et al., 2013; Chen et al., 2016; Katarivas et al., 2017). Subsequently, with the dissolution of Zn, the released Zn ions can be detected, and the released OH^−^ increases the pH value in the HBSS. Based on the Pourbaix diagram, passivated precipitation layers of, i.e., Zn(OH)_2_ and ZnO, are formed on the Zn surfaces, as described in Eq. 3 and Eq. 4 (Chen et al., 2016; Yang et al., 2017). Also, phosphate ions (HPO_4_^2 –^) and carbonate ions (HCO_3_^−^) in the HBSS can further react with free Zn ions to produce Zn phosphate [Zn_3_(PO_4_)_2_·4H_2_O] and Zn carbonate (ZnCO_3_) (Liu X. et al., 2019; Venezuela and Dargusch, 2019), as described in Eq. 5. Meanwhile, some complex degradation products, i.e., hydrozincite [Zn_5_(CO_3_)_2_(OH)_6_] and simonkolleite [Zn_5_(OH)_8_Cl_2_·H_2_O], can be deposited, as previously reported (Venezuela and Dargusch, 2019). Considering the chemical reactions mentioned above, it was confirmed that the elemental composition of degradation products in HBSS was mainly composed of Zn, O, C, P, Ca, and Cl. Additionally, when the samples were immersed in HBSS with FBS, the degradation layers mainly consisted of Zn, C, O, and a small amount of N, K, and Cl, indicating a mixture of inorganic and organic constituents, as previously reported (Zhang et al., 2021a).

$$\mathrm{Anodic}\;\mathrm{reaction}\text{:}\;2\text{Zn}\left(\text{s}\right)\to {2\text{Zn}}^{2+}\left(aq\right)+4e^{-}$$ (1)

$$\mathrm{Cathodic}\;\mathrm{reaction}\text{:}\;{2\text{H}}_{2}O+O_{2}+4e^{-}\to 4\mathrm{OH}$$ (2)

$$\mathrm{Zn}{\left(\mathrm{OH}\right)}_{2}\mathrm{formation}{\text{:Zn}}^{2+}{\mathrm{OH}}^{-}\to \mathrm{Zn}{\left(\text{OH}\right)}_{2}$$ (3)

$$\mathrm{ZnO}\;\mathrm{formation}\text{:}\;Zn^{2+}+{\mathrm{OH}}^{-}\to \mathrm{ZnO}+H_{2}\mathrm{O,}\;\mathrm{Zn}{\left(\mathrm{OH}\right)}_{2}\to \mathrm{ZnO}+H_{2}O$$ (4)

$$\mathrm{Zinc}\;\mathrm{phosphate}\;\mathrm{formation}{\text{:}3\text{Zn}}^{2+}+2OH^{-}+2H_{2}\mathrm{O+2HP}O_{4}^{\mathrm{2-}}\to {\mathrm{Zn}}_{3}{\left(PO_{4}\right)}_{2}\cdot 4H_{2}O$$ (5)

Notably, the degradation layers formed in HBSS with or without FBS exhibited distinct patterns. Relatively thin degradation layers were formed on the surfaces in HBSS with FBS, indicating that the serum protein in FBS might delay the Zn degradation process. Also, according to the weight loss results, the degradation rates of Zn and Zn-Cu-Fe in the HBSS were significantly higher than their counterpart in the HBSS with FBS. To date, the effects of serum on the degradation behavior of Zn and its alloys have been controversially discussed since experimental parameters were considered independently. Although FBS was used to provide serum proteins in simulated body fluids, it is still unclear how the serum affects degradation mechanisms. Li et al. (Li et al., 2019a) demonstrated that FBS in cell culture media could effectively accelerate initial Zn ion release (the first 24 h), probably because protein attachment prevented the formation of the initial passivated layer.

On the contrary, Zhang et al. (Zhang et al., 2021a) reported that pure Zn and Zn-Cu alloy were immersed in human peripheral blood, human serum, and Dulbecco’s phosphate-buffered saline (DPBS) for 28 days. The results showed that the Zn-based metals in the blood and serum had uniform degradation behavior, while severe localized corrosion could be found in DPBS, verified by the main finding of this study. The seemingly conflicting results of the degradation process might be explained by the different stages of Zn degradation under exposure to serum protein (Liu L. et al., 2019; Dong and Virtanen, 2022). Accordingly, we assume that organic layers on the Zn surfaces could prevent the chloride from diffusing into and attacking the matrix, retarding the degradation.

In general, the degradation rates of Zn-based alloys are higher than pure Zn, principally due to the micro-galvanic effect between the Zn matrix (anode) and its secondary phase (cathode), as previously reported (Li et al., 2018a; Sagasti et al., 2019; Capek et al., 2021). Nonetheless, in the present study, the degradation rate of pure Zn was significantly higher than that of the Zn-Cu-Fe alloy, probably contributing to its microstructure characteristics. A recent study reported that a hot-extruded Zn-2Cu-0.5Zr alloy had a lower degradation rate than pure Zn due to its refined grains (Tan et al., 2021). Our previous study also demonstrated that hot-extruded Zn-Cu-Fe alloy possessed a relatively well-distributed FeZn_13_ secondary phase in the Zn matrix (Figures 10A,C). Therefore, we postulate that the refined grains of the Zn-Cu-Fe alloy resulted in a more uniform degradation mode in the Zn matrix than pure Zn. As shown in Figures 10B,D, the Zn matrix exhibited coarse grains, and the degradation product layer formed on the surface was loose and porous, making it difficult to cover the Zn matrix completely. The chloride ion in Hank’s solution can directly attack the matrix through the defects within the degradation product layer (Figure 10B), promoting the corrosion of the matrix and accelerating the formation of local corrosion galvanic cells (Yue et al., 2019). After adding an alloying element, the matrix phase was further refined (Figure 10C). The corrosion product layer on the surface was more uniform and denser, and the degradation layer showed more integrity (Figure 10D). The relatively complete passivated layer on the Zn-Cu-Fe can prevent its localized corrosion, probably delaying the degradation process of the Zn-Cu-Fe alloy. It should be noted that the *in vivo* degradation of the Zn-Cu-Fe alloy might be more complicated due to the intervention of the host’s immune system. Medical imaging techniques (Zhang et al., 2011) might be useful for a dynamic examination in future animal studies.

### Cytotoxicity and Hemocompatibility of Zn-Cu-Fe Alloy

Biocompatibility refers to a biomaterial performing with an appropriate host response, which is the most important prerequisite for clinical applications. Zn-based metals have emerged as a new generation of biodegradable materials, and their biological safety has attracted increasing attention. Zn ion is the primary degradation product released from Zn-based degradable metals. The ionic Zn is well known and is regarded as the most abundant transition metal ion in the human body (Tapiero and Tew, 2003). The Zn ion plays a significant role in various physiological functions, including the development and maintenance of bone health (Yamaguchi, 1998), protection against coronary artery disease (Little et al., 2010), and even the integrity of the immune function (Dardenne, 2002). Notably, an independent cross-sectional study demonstrated that human serum zinc levels are significantly associated with increased bone mineral density in the total spine and total femur (Qu et al., 2020). Zinc-based biomaterials were developed and investigated to treat bone-related diseases considering the biological advantages of zinc. Nonetheless, excessive Zn exposure led to detrimental effects on the organs (Chasapis et al., 2012). At the cellular level, excessive Zn ions can inhibit the electron transport mechanism in uncoupled mitochondria (Kleiner, 1974). In addition, Zn ions exert biphasic effects on cell viability, adhesion, and proliferation (Li et al., 2020). High serum zinc levels were associated with increased risks of diabetes mellitus and cardiovascular diseases (Qu et al., 2020). Therefore, the biosafety of Zn-based alloys and their degradation products must be carefully identified and evaluated.

In this study, based on the ISO standards (10993-5 and -12), an extract test was used for the cytotoxicity evaluation of Zn and Zn-Cu-Fe. The results showed that Zn and Zn-Cu-Fe had consistent cytotoxic effects on macrophages, osteoblast precursor cells, and endothelial cells, i.e., the undiluted original sample extracts had apparent cytotoxic effects while the toxic effects decreased after the extracts were diluted. This overall tendency is in line with the cytotoxicity results of previous investigations, including zinc-copper alloys (Tang et al., 2017; Lin et al., 2020), zinc-magnesium alloys (Kubasek et al., 2016; Shen et al., 2016), zinc-germanium alloys (Tong et al., 2018), and zinc-silver alloys (Li et al., 2018a; Sagasti et al., 2019). In principle, the extract test investigates the influence of released degradation products on cell viability, principally attributed to two elements: released Zn-based degradation products during the extraction process and cellular tolerance capability (Wang et al., 2015; Li et al., 2018b). In undiluted extracts, Zn ion concentration might be beyond the related cellular tolerance, leading to apoptosis, as previously reported (Tang et al., 2017; Lin et al., 2020). Previous studies also reported that undiluted extracts of Zn-based alloys exhibited no apparent cytotoxic effects (Li et al., 2019b; Zhang et al., 2021b). The inconsistent results can be attributed to the absence of serum in extraction vehicles. A previous study reported that a serum-supplemented extract medium could accelerate the degradation, leading to additional cytotoxic effects (Li et al., 2019a). Nonetheless, these conflicting results should not be overestimated. The ISO standards are designed to evaluate bioinert materials, neglecting the degradability of BMs (Bruinink and Luginbuehl, 2011; Li et al., 2018b). At least a six-fold dilution of the extracts for Mg-based alloys was recommended for the cytotoxicity evaluation to mimic the physiologic metabolism and elimination *in vivo* (Fischer et al., 2011; Wang et al., 2015). Herein, the Zn-Cu-Fe alloy exhibited good cytotoxicity results. In response to a specific stimulus, mature macrophages can transfer into different phenotypes, which indicates that mature macrophages have a self-renewal capability similar to that of stem cells (Sieweke and Allen, 2013). The regulation of zinc homeostasis has also been found to be important to the maintenance of macrophage function in recent years. The dysregulation of zinc homeostasis in macrophages resulted in an abnormal inflammatory response as well as impaired phagocytosis (Gao et al., 2018). Meanwhile, zinc deficiency leads to the abnormal secretion of immune factors in response to specific infections. Furthermore, oxidative stress caused by altered levels of zinc can cause the innate immune system to malfunction during acute inflammation (Roy et al., 2014; Summersgill et al., 2014; Wong et al., 2015; Pyle et al., 2017). Previous studies demonstrated that zinc deficiency is capable of inducing apoptosis through a mitochondria-mediated pathway in osteoblastic cells (Guo et al., 2012).

Under physiologic conditions, most CMF implant materials initially come into contact with human blood components after implantation. In fact, the material surface and released degradation products can trigger a series of blood cell reactions, including serum protein absorption, blood cells’ adhesion/activation, coagulation, and complement system cascade reactions. (Weber et al., 2018). In the present study, a modified Chandler-Loop model was used to evaluate the blood compatibility of Zn-Cu-Fe, and erythrocytes, platelets, and leukocytes were investigated. Regarding erythrocytes, the results indicated that Zn and Zn-Cu-Fe did not dramatically decrease erythrocyte counts, and their hemolysis was not affected adversely under the circulation conditions (Figure 7), consistent with previous findings that Zn and Zn-based alloys have relatively low induction of hemolysis (<5%) in static or dynamic models (Yin et al., 2019; Zhang et al., 2021a). Concerning platelets and their activation, platelet counts exhibited no apparent differences in the present study. However, the concentration of β-TG was significantly increased, as a marker for granule release, indicating platelet activation. Also, an increased tendency of TAT concentration was observed, implying a potential risk of thrombin formation. These findings are inconsistent with the previous results obtained from the static test, which indicated that the prothrombin time and partial thromboplastin time of Zn and Zn-based alloys were significantly prolonged (Yin et al., 2019). This discrepancy is probably caused by the increased degradation release of Zn-Cu-Fe under dynamic conditions, leading to the activation of coagulation. Notably, leukocytes are believed to play a critical role in the innate immune system. Also, leukocyte counts indirectly reflect the inflammatory response triggered by an implant (Jaffer and Weitz, 2019). In the Chandler-Loop model, the Zn-Cu-Fe did not adversely affect the leukocyte number under the dynamic condition. Released PMN-elastase complexes are a highly sensitive marker of inflammation. There were no significant differences in PMN-elastase complexes between different groups. However, the tendency for upregulating Sc5b-9 could be observed, indicating that the early inflammation was probably induced by Zn and Zn-Cu-Fe. Regarding the implant applications with a bony environment, Sc5b-9 positively affected bone regeneration, which not only did upregulate the osteoprotegerin expression but inhibited the osteoclast activity as well (Corallini et al., 2009). Taken together, the results indicated that the Zn-Cu-Fe alloy had acceptable cytocompatibility and hemocompatibility.

## Conclusion

The present study investigated the *in vitro* biodegradability, cytocompatibility, and hemocompatibility of a hot-extruded Zn-Cu-Fe alloy as a potential implant material for craniomaxillofacial reconstructive surgery. The immersion tests indicated that incorporating FBS into HBSS could decrease the degradation rate of the Zn-Cu-Fe alloy, probably due to the formation of a protein layer. Compared with pure Zn, an apparent decrease in the degradation process could be found following the incorporation of alloying elements into the Zn matrix. Although the undiluted sample extracts had apparent cytotoxic effects, RAW264.7, HUVEC, and MC3T3-E1 cells cultured in the diluted extracts exhibited relatively high cell viability, indicating good cytotoxic effects. Furthermore, the Chandler-Loop test demonstrated that the Zn-Cu-Fe alloy did not adversely affect blood cell counts while the coagulation and complement systems were upregulated. In summary, the hot-extruded Zn-Cu-Fe alloy exhibited good performance in terms of biodegradability, cytocompatibility, and hemocompatibility and might be a promising degradable material for CMF implants.

## Data Availability

The raw data supporting the conclusions of this article will be made available by the authors, without undue reservation.
